# Behavioral modifications by a large-northern herbivore to mitigate warming conditions

**DOI:** 10.1186/s40462-020-00223-9

**Published:** 2020-10-15

**Authors:** Jyoti S. Jennewein, Mark Hebblewhite, Peter Mahoney, Sophie Gilbert, Arjan J. H. Meddens, Natalie T. Boelman, Kyle Joly, Kimberly Jones, Kalin A. Kellie, Scott Brainerd, Lee A. Vierling, Jan U. H. Eitel

**Affiliations:** 1 Department of Natural Resources and Society, University of Idaho, Moscow, ID USA; 2 Wildlife Biology Program, Department of Ecosystem and Conservation Science, W.A. Franke College of Forestry and Conservation, University of Montana, Missoula, MT USA; 3 College of the Environment, University of Washington, Seattle, WA USA; 4 Department of Fish and Wildlife Sciences, University of Idaho, Moscow, ID USA; 5 School of the Environment, Washington State University, Pullman, WA USA; 6 Lamont-Doherty Earth Observatory, Columbia University, Palisades, NY USA; 7 National Park Service, Gates of the Arctic National Park and Preserve, Fairbanks, AK USA; 8 Alaska Department of Fish and Game, 1800 Glenn Hwy #2, Palmer, AK USA; 9 Alaska Department of Fish and Game, Division of Wildlife Conservation, 1300 College Rd, Fairbanks, Alaska, USA; 10 Department of Forestry and Wildlife Management, Inland Norway University of Applied Sciences, Evenstad, Norway; 11 McCall Outdoor Science School, University of Idaho, McCall, ID USA

**Keywords:** Climate change, Behavioral thermoregulation, Thermal stress, Ambient temperature, Habitat selection, Wildlife, *Alces alces*

## Abstract

**Background:**

Temperatures in arctic-boreal regions are increasing rapidly and pose significant challenges to moose (*Alces alces*), a heat-sensitive large-bodied mammal. Moose act as ecosystem engineers, by regulating forest carbon and structure, below ground nitrogen cycling processes, and predator-prey dynamics. Previous studies showed that during hotter periods, moose displayed stronger selection for wetland habitats, taller and denser forest canopies, and minimized exposure to solar radiation. However, previous studies regarding moose behavioral thermoregulation occurred in Europe or southern moose range in North America. Understanding whether ambient temperature elicits a behavioral response in high-northern latitude moose populations in North America may be increasingly important as these arctic-boreal systems have been warming at a rate two to three times the global mean.

**Methods:**

We assessed how Alaska moose habitat selection changed as a function of ambient temperature using a step-selection function approach to identify habitat features important for behavioral thermoregulation in summer (June–August). We used Global Positioning System telemetry locations from four populations of Alaska moose (*n* = 169) from 2008 to 2016. We assessed model fit using the quasi-likelihood under independence criterion and conduction a leave-one-out cross validation.

**Results:**

Both male and female moose in all populations increasingly, and nonlinearly, selected for denser canopy cover as ambient temperature increased during summer, where initial increases in the conditional probability of selection were initially sharper then leveled out as canopy density increased above ~ 50%. However, the magnitude of selection response varied by population and sex. In two of the three populations containing both sexes, females demonstrated a stronger selection response for denser canopy at higher temperatures than males. We also observed a stronger selection response in the most southerly and northerly populations compared to populations in the west and central Alaska.

**Conclusions:**

The impacts of climate change in arctic-boreal regions increase landscape heterogeneity through processes such as increased wildfire intensity and annual area burned, which may significantly alter the thermal environment available to an animal. Understanding habitat selection related to behavioral thermoregulation is a first step toward identifying areas capable of providing thermal relief for moose and other species impacted by climate change in arctic-boreal regions.

## Background

Global temperatures are drastically increasing [36], which directly affect animal behavior and fitness [9, 88, 91]. When ambient temperatures rise above an animal’s thermal neutral zone, they use physiological and behavioral mechanisms to dissipate heat and mitigate thermal stress. For instance, additional energy may be spent to augment the cardiovascular and respiratory systems enabling evaporative cooling but may also lead to dehydration [16, 54, 73]. Consequentially, increases in ambient temperature may contribute to a negative energy balance within an animal [5, 85, 87]. Energetic requirements of mammals vary by season and traits (e.g., body mass, lactation). Summer is an important season for mammals as they need to recover from winter food deficits, lactate and rear young, and store fat [14, 75, 85]. Climate change puts further stress on these important activities, which may, in turn, limit the ability of mammals to meet energetic requirements for reproduction and survival [25, 50, 90]. Recent work suggests that large-bodied mammals respond more strongly to climate change, when compared to smaller-bodied mammals, through contraction or expansion of elevational ranges and also experience increased extinction risk [53].

Moose (*Alces alces*) are an important, large-bodied mammal vulnerable to increasing temperatures because they are well-adapted to cold climates [73, 76]. Moose also act as ecosystem engineers, by regulating forest carbon and structure, below ground nitrogen cycling processes, and predator-prey dynamics [12, 15, 48, 55]. According to the seminal physiological study by Renecker and Hudson [73], moose reached their upper critical temperature threshold at 14 °C in summer where they increased their heart and respiration rates, while open-mouthed panting began at 20 °C. However, recent works call these thresholds into question and suggest there is no static temperature threshold where free-ranging moose become heat stressed [83, 84]. Similarly, behavioral changes are often observed at temperatures that exceeds the upper critical summer threshold proposed by Renecker and Hudson [73] [11, 56].

Behavioral alterations elicited by changes in temperature influence both resource selection patterns and movement rates. For example, previous studies showed that during hotter periods, moose displayed stronger selection for riparian or wetland habitats [74, 80], taller and denser forest canopies that provide thermal cover [20, 56, 88], and minimized exposure to solar radiation [54]. Additionally, moose may also decrease their activity and movement rates in response to warmer daytime temperatures [58, 80].

Moose thermoregulatory behaviors are indeed a ‘hot topic’ in applied ecology because of rising temperatures related to climate change and their important ecosystem role (e.g., [56, 58, 80]). However, most previous studies occurred in Europe or the southern end of moose range in North America [50, 56, 88]. Understanding whether ambient temperature elicits a behavioral response in high-northern latitude (i.e., ≥ 60°N) moose populations in North America may be increasingly important as these arctic-boreal systems have been warming at a rate two to three times the global mean [2, 36, 77, 95] and current projections anticipate continued increases in temperature [36, 51]. Thus, it is important to explore how movement patterns of moose, a heat-sensitive large-bodied mammal, are influenced by changes in temperature at the northern extent of their range.

**Fig. 1:**
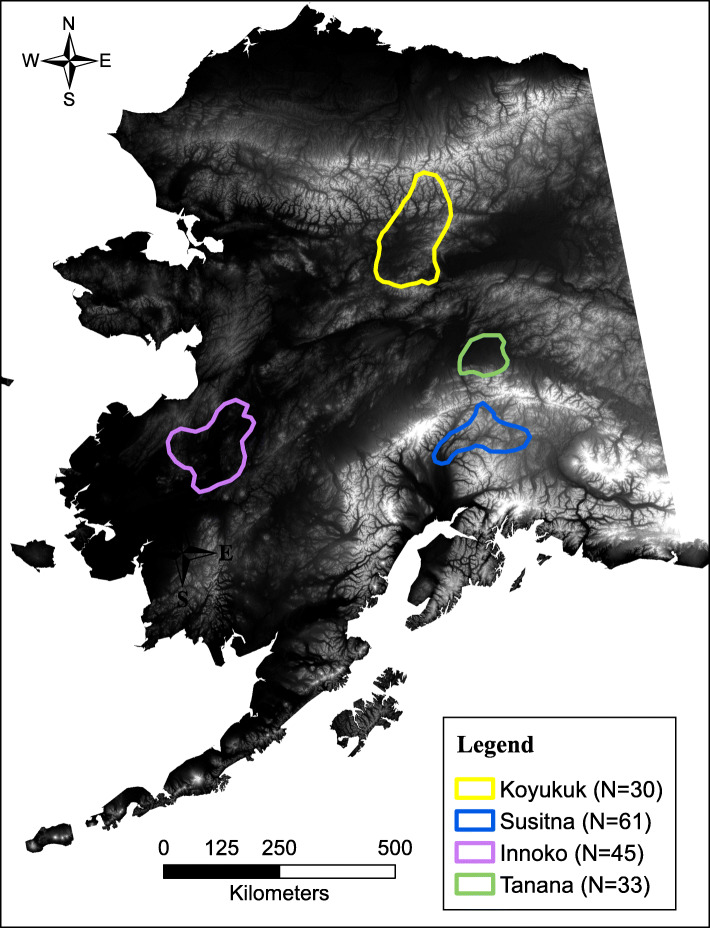
Moose (*Alces alces gigas*) study area locations in four distinct ecoregions of Alaska, USA. In total, 169 moose were included in these analyses (111 females; 58 males)

Accordingly, our study objective was to assess Alaska moose (*Alces alces gigas*) habitat selection as a function of ambient temperature. We tested the hypothesis that moose modified resource selection in response to ambient temperature as predicted by physiological models. To accomplish this, we used Global Positioning System (GPS) -telemetry locations from four Alaska moose populations (*n* = 169 moose; Fig. 1 & Table 1) from 2008 to 2016 that were located in four unique ecoregions [65]. We combined moose GPS locations with remotely sensed products important to thermoregulatory behaviors. We analyzed only summer months (June–August) because of their importance in moose life history and because thermal stress is most likely to occur in summer [23, 88]. Each population was analyzed independently and separated into male and female subsets because fine-scale movements vary by sex and local habitat characteristics [41, 43, 49]. We predicted that Alaska moose exhibit a detectable behavioral response to increasing summer temperatures, and, that as temperature increased, moose would select for cooler locations, such as thermal refugia provided through increased canopy cover, areas closer to water, and/or low exposure to solar radiation.

**Table 1:** Summaries of Alaska moose (*Alces alces gigas*) Global Positioning System (GPS) datasets by study area. Information on the number of fixes and the fix success rate are specific to summer (June 1 – August 31). The number of clusters for each population-sex partition refer to the unique combination of individual-year, which were used in our conditional logistic regression models as a clustering variable for estimating robust variance estimates using generalized estimating equations

Dataset	Number of moose	Number females (clusters)	Number males (clusters)	Years	Fix rate (hours)	Fix success	Number of fixes
Koyukuk	30	19 (45)	11 (22)	2008–2013	8	91%	F- 11,324
							M- 3949
Susitna	61	38 (71)	23 (36)	2012–2016	8	98%	F- 14,984
							M-6003
Innoko	45	21 (63)	24 (65)	2010–2014	4^a^	95%	F- 2319
							M- 1987
Tanana	33	33 (145)	0	2011–2016	3.5^a^	99%	F- 21,530
**Totals:**	169	111	58	–	–	96%	F-50,157
							M- 11,939

^a^ data with less than 8-h fix rates were aggregated to near 8-h fix rates

## Methods

### Study areas

All four study areas span a mixture of subarctic and arctic boreal forest vegetation including black spruce (*Picea mariana*), alders (*Alnus* spp.), willows (*Salix* spp.), Alaska birch (*Betula neoalaskaa*), white spruce (*Picea glauca),* quaking aspen (*Populus tremuloides*), and balsam poplar (*Populus balsmifera*). The upper Koyukuk region located in the Brooks Mountain Range (Fig. 1) is rugged and varies from 500 to 2600 m above sea level [1]. Wildfire is common in this region, which experiences strongly continental climate patterns where summers are short, but temperatures can exceed 30 °C [41]. Average daily summer (June–August) temperature ranged from 7.5 °C to 15 °C from 1986 to 2016 [64]. The Tanana Flats region is located south of Fairbanks, where the alluvial plane from the Alaska Mountain Range slopes northward making meandering rivers and oxbow lakes common [1]. Elevation ranges from 0 to 700 m, however the highest elevations occurred in the northern portion of the Alaska Mountain Range [1]. The Tanana region experiences dry-continental climate, and average daily summer temperature ranged from 11 °C to 19.5 °C from 1986 to 2016 [64]. The Innoko region lies in southwest Alaska and includes a portion of the lower Yukon River. Meandering waterways, oxbow lakes and floods are common in the lowlands while upland areas experience more wildfire disturbance [67]. Elevation varies little (30–850 m) and average daily summer temperatures ranged from 9.5 °C to 17.5 °C from 1989 to 2016 [64]. The Susitna moose range lies south of Alaska Mountain Range, and is characterized by numerous wetlands, hilly moraines, black spruce woodlands, and mountains. Elevation varies widely from 400 to 3500 m. This region is primarily located in temperate-continental climate, with some exposure to temperate coastal climates in the southern portion of the range [1]. Average daily summer temperatures ranged from 11.5 °C to 19 °C from 1988 to 2016 in this region [64].

### Moose data

All capture protocols and handling protocols adhered to the Alaska Animal Care and Use Committee approval process (#07–11) as well as the Institutional Animal Care and Use Committee Protocol (#09–01). Moose in all regions were darted from helicopter (Robison R-44) and injected using carfentanil citrate (Wildnil® Wildlife Pharmaceuticals, Incorporated, Fort Collins, CO) and xylazine hydrochloride (Anaset®; Lloyd Laboratories, Shenandoah, IA). Moose were instrumented with GPS radio-collars with three and a half to eight-hour fix rates (Table 1). Specifically, moose were fitted with the following collars from Telonics Inc. (Telonics, Mesa, AZ): Koyukuk – GW-4780, Tanana –TGW-4780-3, Susitna – TGW-4780-2, Innoko –CLM-340.

### Statistical analyses

#### Habitat selection

We used a step-selection function (SSF) to assess moose behavioral responses to changing temperatures. SSF’s model habitat selection in a used-available design that accounts for changing availability of resources at any point in time [27, 86]. We aggregated moose datasets to a near eight-hour fix rate to enable regional comparisons of behavior (Table 1). We chose this modeling framework because it allows for assessments of fine-scale habitat selection, and the effect of temperature on large herbivore movement behavior are most pronounced at fine to intermediate spatial and temporal scales [89]. To sample availability, we generated ten-paired available locations based on empirical distributions of an individual’s step length and turning angles between sampling intervals, which were estimated using the “ABoVE-NASA” R package [29]. We used conditional-logistic regression (CLR, [35]) in the “survival” R package [82] to compare each used location with the concurrent available locations at the same point in time and space (i.e., one stratum contained one used point and ten randomly generated available points). The equation can be written as:

1 $$ w\ast \left(\mathrm{x}\right)=\frac{\mathit{\exp}\left(\beta 1\mathrm{x}1+\beta 2\mathrm{x}2+\dots +\beta \mathrm{nxn}+e\right)}{1+\mathit{\exp}\left(\beta 1\mathrm{x}1+\beta 2\mathrm{x}2+\dots +\beta \mathrm{nxn}+e\right)} $$

where w*(x), the relative probability of selection, is dependent on habitat covariates *X*_1_ through *X*_n_, and their estimated regression coefficients *β1* to *βn*, respectively. Steps with higher w*(x) indicate a greater chance of selection. CLR compares strata (i.e., one used point and ten available points) individually, which enabled us to assess selection of fine-scale habitat features rather than broader-scale landscape characteristics [6]. We did not directly incorporate random effects into our SSF models as the analytical techniques for doing this are sparse and often computationally prohibitive for complex model sets [61]. In our models, we would have a needed to incorporate a random effect of individual for each covariate in the model – the equivalent of random slopes. We believe this would likely have led to convergence issues as our models are already complex (see section regarding temperature interaction terms). Instead, we fit our CLR models with generalized estimating equations (GEE) using a clustering variable of “animal-year” to split the data into statistically independent clusters. This allowed us to account for lack of independence between steps within an individual for a given summer, and provided unbiased (i.e., robust) variance estimates provided there are at least 20 independent clusters and preferably 30 [71]. Our data all had at least 20 unique animal year clusters, and all but one had greater than 30 (Table 1).

#### Habitat covariates

We obtained temperature estimates from the North American Regional Reanalysis (NARR) as opposed to weather stations. NARR provides a suite of highly-temporally dynamic (eight times daily; 32 km) set of meteorological variables [57]. We annotated NARR temperature estimates using the environmental-data automated track annotation (Env-DATA) system available from Movebank [21]. To ensure accuracy of these temperature estimates, we performed a validation exercise on the two populations of moose which included temperature sensors on their collars (Innoko and Koyukuk). We found a moderate relationship between the two (Supplementary material (S)1; *R*^*2*^ = 0.47–0.58, RMSE = 3.88–4.43 °C). NARR temperature estimates represent an ambient, neighborhood temperature, allowing us to investigate how moose respond to ambient variation in temperature via fine-scale selection for environmental characteristics that are likely to create cooler microclimates. We excluded ambient temperature as a main effect within CLR models because it did not vary within strata, and only included it as an interaction term with other covariates.

Moose may move to areas that provide thermal cover when temperatures increase such as denser canopied forests [56]. In our models, a United States Geological Survey (USGS) percent canopy product for 2010 (30 m cell size, [31]) was used as an index of thermal cover. Moose use canopy cover for purposes other than thermoregulation such as predator avoidance [85]. However, by considering the interaction between temperature and canopy cover, it is likely that we captured behavioral thermoregulation in our models.

We assessed the importance of water habitats in behavioral thermoregulation using a distance-to-water covariate. We estimated this covariate from Pekel et al.’s [68] percent global surface water map, which quantified global surface water from 1984 to 2015. We used the R “raster” package [34] to estimate the Euclidian distance of the nearest water pixel (30 m cell size) from a given moose location. Elevation estimations (in meters) were extracted from the ArcticDEM (version 6, 5 m cell size [69];). The solar radiation index (SRI [46];) was estimated mathematically as a function of latitude, aspect, and slope using the “RSAGA” package [8] – which were derived from the ArcticDEM, with the resultant values representing the hourly extraterrestrial radiation striking an arbitrarily oriented surface [46].

We chose to consider only continuous covariates as predictors to represent habitat as dynamic and continuous (sensu [17]). Covariates were standardized by dividing them by two times their standard deviation [28], allowing coefficients to be directly comparable across models. Collinearity was assessed using Pearson correlation coefficients, if correlation coefficients between predictors exceeded 0.70 we excluded collinear metrics from being present in the same model [22].

#### Two-way temperature interactions

We considered both linear and nonlinear interactions between habitat covariates and ambient temperature as nonlinear processes are widespread in ecology particularly in response to climate change [13, 92]. In total, three model variants for each population-sex partition were considered: (1) a *base model* that included habitat covariates as described above with no interaction terms or consideration of temperature, (2) *linear interaction models* where habitat covariates sequentially interacted with temperature linearly, and (3) *spline interaction models* where habitat covariates sequentially interacted nonlinearly with temperature using natural cubic splines. Because nonlinear terms are at risk of overfitting models, we constrained any nonlinear relationships explored in the spline interactions to two or three knots in CLR models using the “splines” package [72].

#### Habitat selection model evaluation and validation

We evaluated model fit for each population-sex partition using the quasi-likelihood under independence criterion (QIC [66];) because it is well suited for case-control models [19]. Finally, predictive ability of model variants were assessed using leave-one-out cross validation (LOOCV), which is a k-fold cross validation variant [7] where each individual animal is sequentially left out and predicted based on the remaining data. Mean Spearman rank coefficients were used to determine the predictive ability of model variants. For each population-sex partition, the model with the highest correlation coefficients from LOOCV and lowest QIC was considered the best. All spatial processing and statistical analyses were conducted in the statistical software R version 3.6.1 [72].

## Results

In total, seven base, 28 linear interaction, and 28 spline interaction models were estimated. For the sake of parsimony, only the most biologically significant results are presented and summarized by sex and population. Elevation was collinear with distance-to-water in the Innoko population, we retained the latter because of its known importance in moose ecology [74, 80]. In all but one case (Koyukuk males, S2), spline-based models where percent canopy interacted with temperature outperformed linear interaction and base models and are thus the only models discussed (Tables 2 and 3). In contrast to the strong habitat selection responses of moose for canopy cover, we did not find evidence for other behavioral thermoregulation strategies. For example, we found no support that Alaska moose altered resource selection with increasing summer temperatures in response to topography (i.e., more northerly, cooler slopes), elevation (with the exception of one population, S2), nor hydrology (i.e., by selecting to be closer to water).

**Table 2:** Model evaluation (QIC) and cross validation (LOOCV) for female moose organized by population. Base models contain no temperature covariates, while spline models incorporate nonlinear interactions between a given covariate and ambient temperature. In this case, “Spline %can2” refers to percent canopy interacted with ambient temperature with two spline segments, while “Spline %can3” refers to percent canopy interacted with ambient temperature with three spline segments. Decreases in QIC indicate a better model fit while increases in LOOCV indicate more predictive ability

	Koyukuk		Susitna		Innoko		Tanana	
	*Base*	*Spline %can2*	*Base*	*Spline %can2*	*Base*	*Spline %can2*	*Base*	*Spline %can3*
QIC	47,070	46,918	70,707	70,423	73,361	73,184	102,854	102,746
ΔQIC	–	− 152	–	−284	–	− 177	–	−108
LOOCV	68%	69%	62%	64%	60%	63%	36%	46%
ΔLOOCV	–	+ 1%	–	+ 2%	–	+ 3%	–	+ 10%

Note: %can = percent canopy cover

**Table 3:** Model model evaluation (QIC) and cross validation (LOOCV) for male moose summary of organized by population. See additional descriptors in Table 3

	Koyukuk		Susitna		Innoko	
	*Base*	*Spline %can2*	*Base*	*Spline %can2*	*Base*	*Spline %can2*
QIC	18,583	18,529	27,919	27,777	62,946	62,849
ΔQIC	–	−54	–	− 142	–	−97
LOOCV	42%	45%	57%	62%	50%	56%
ΔLOOCV	–	+ 3%	–	+ 5%	–	+ 6%

### Females

The best fit spline models across all four populations occurred when percent canopy interacted with temperature using two to three knots. These spline interaction models had significant improvements in model fit compared to both the base models (ΔQIC = − 108 to − 284; Table 2) and the linear interaction models (not shown). Cross validation scores for spline interaction models experienced small to moderate improvements when compared to the base model (ΔLOOCV = + 1% to + 10%; Table 3).

**Fig. 2:**
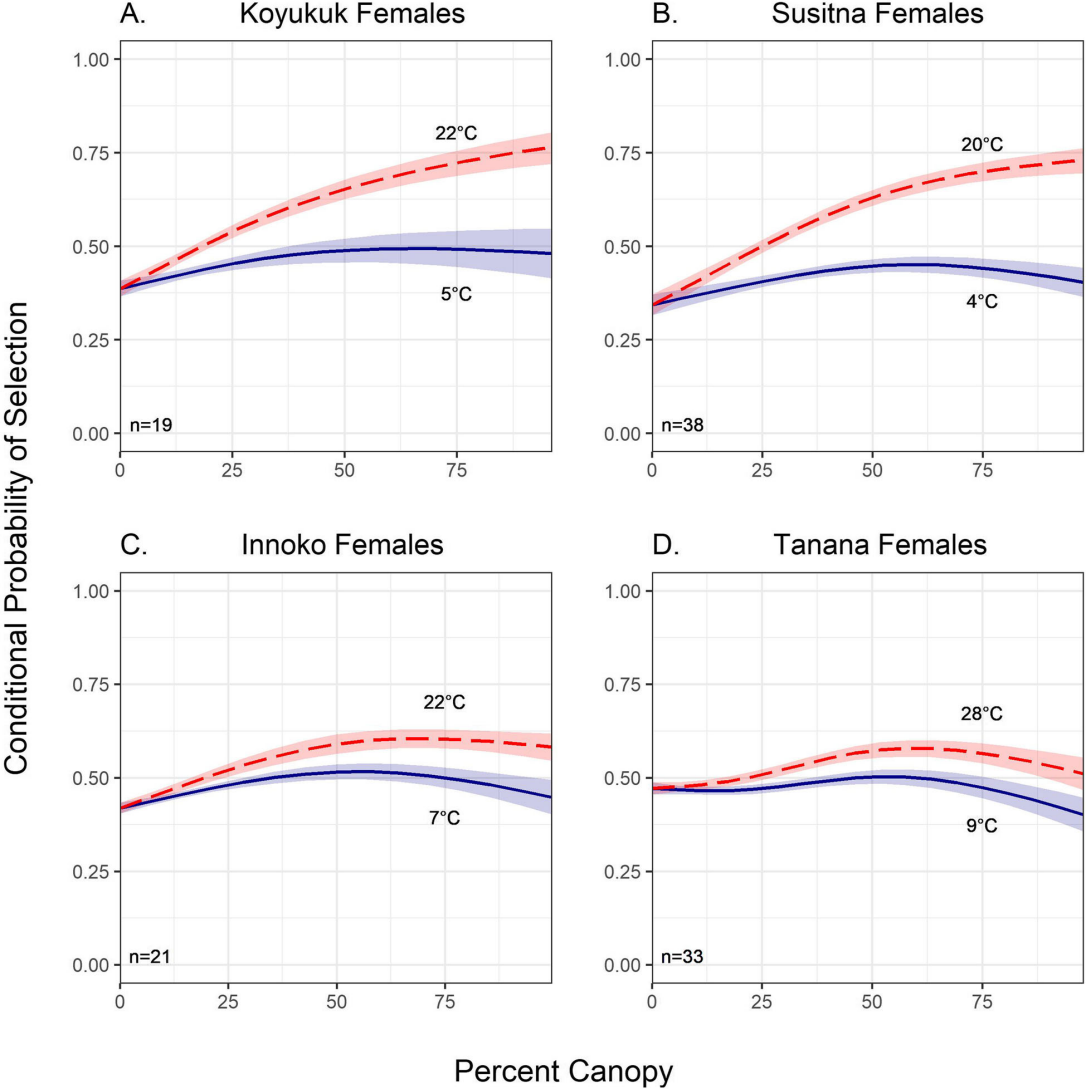
Conditional probability of selection of spline-based thermal cover as a function of temperature for Alaskan female moose by region in summer months (June–August). We used natural splines with two to three degrees of freedom to represent the relationship between canopy cover and temperature. The probability of selection of denser canopy increased significantly with temperature during summer for all four regions, where red lines indicated the 90% temperature percentiles of experienced temperature and the blue lines indicate the 10% temperature percentiles experienced temperature by region. Shaded bands represent a 95% confidence interval. Plots were created in the ‘ggplot2’ R package [94]

In summer, female moose in all four regions selected for increased canopy cover nonlinearly as temperature increased (Fig. 2; S3). However, the magnitude of the selection response to thermal cover was most pronounced in the most southerly region (Susitna; β_%canopy1_ = 33.90, *p* < 0.001; β_%canopy2_ = 20.09, *p* < 0.001; Table 4) as well as the most northerly region (Koyukuk; β_%canopy1_ = 24.91, *p* < 0.001; β_%canopy2_ = 20. 03, *p* < 0.001). Although the effect of canopy cover was reduced in both the Innoko moose (β_%canopy1_ = 14.82, *p* < 0.001; β_%canopy2_ = 9.01, *p* < 0.001) and the Tanana moose (β_%canopy1_ = 4.71, *p* < 0.001; β_%canopy2_ = 8.97, β_%canopy3_ = 7.70, *p* < 0.001), both populations still revealed highly statistically significant results indicating female moose selected nonlinearly for increased canopy cover as temperature increased.

**Table 4:** Best habitat selection models by population for female moose (*Alces alces gigas*) in Alaska from the step-selection function analysis. The best models across all four populations occurred when percent canopy interacted with temperature nonlinearly and are presented here. Natural spline (sp) predictors, where percent canopy interacted with temperature, have coefficients estimated for each line segment. Therefore, numbers one through three in the spline predictor terms represent an individual line segment. Only one of four populations (Tanana) has a third set of coefficients. In the Innoko population, elevation was collinear with distance-to-water and was thus excluded. All predictors were standardized by dividing by two times their standard deviation, making coefficients directly comparable. Robust standard errors are reported

Predictor	Population			
	Koyukuk	Susitna	Innoko	Tanana
	Coefficient (SE)	Coefficient (SE)	Coefficient (SE)	Coefficient (SE)
Elevation	0.09 (0.16)	−1.21 (0.25)^b^	NA	0.28 (0.39)
sp(Percent Canopy x Temperature) 1	24.91 (3.7)^b^	33.90 (3.1)^b^	14.82 (3.32)^b^	4.71 (1.07)^b^
sp(Percent Canopy x Temperature)2	20. 03 (3.1)^b^	20.09 (1.9)^b^	9.01 (2.14)^b^	8.97 (2.22)^b^
sp(Percent Canopy x Temperature)3	NA	NA	NA	7.70 (1.97)^b^
Percent Canopy	−13.90 (2.2)^b^	−16.60 (1.6)^b^	−7.90 (1.92)^b^	−4.80 (1.21)^b^
Solar Radiation Index	0.02 (0.02)	0.003 (0.02)	−0.18 (0.02)^b^	−0.0006 (0.02)
Distance-to-Water	−0.66 (0.3)^a^	− 0.48 (0.09)^b^	−0.22 (0.16)	− 0.09 (0.07)

^a^0.05; **0.01; ^b^0.001

Female moose in the Koyukuk and Susitna regions also showed an increased affinity for water demonstrated in the significant negative beta coefficients for the “distance-to-water” predictor (Table 4), suggesting that moose in these regions preferred to be closer to water. Additionally, we observed additional selection behaviors in the Innoko and Susitna female moose. Female moose in the Innoko population showed an avoidance of areas of high solar radiation (β_SRI_ = − 0.18, *p* < 0.001), while females in the Susitna population showed an avoidance of higher elevation locations (β_elevation_ = − 1.21, *p* < 0.001), but these results were independent of temperature.

### Males

For males, the best fit spline models in the Susitna and Innoko populations were also from percent canopy interacted with temperature (ΔQIC = −142 and − 97 respectively; Table 3). For the Koyukuk males, the best fit spline model came from elevation interacted with temperature (S2), but males in this region also saw improved model fit from percent canopy interacted with temperature (ΔQIC = − 54). Cross validation scores for spline interaction models (percent canopy interacted with temperature) in all three male populations experienced small to moderate increases when compared to the base model (ΔLOOCV = + 3% to + 6%).

**Fig. 3:**
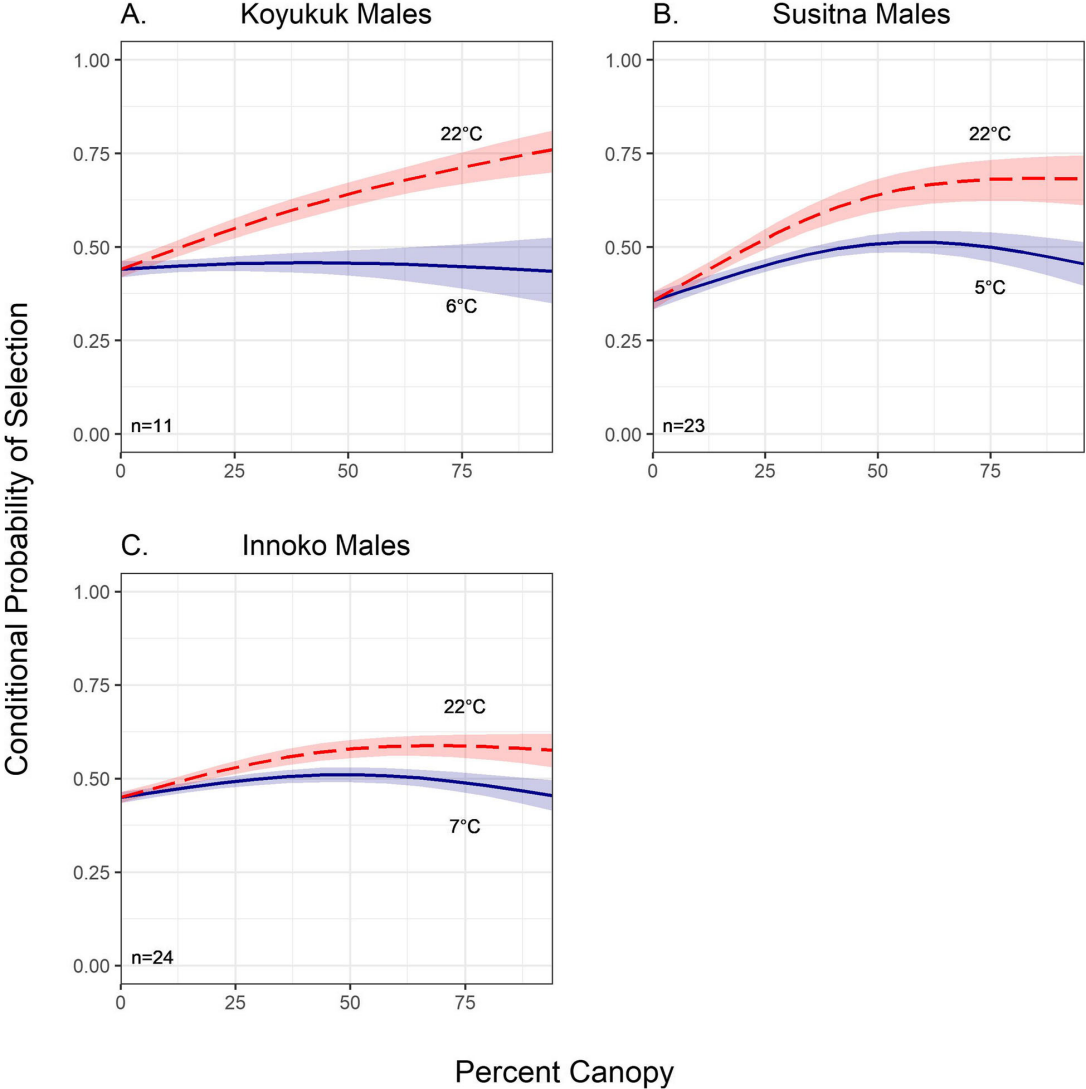
Conditional probability of selection of spline-based thermal cover as a function of temperature for Alaskan male moose by region in summer months (June–August). We used natural splines with two to three degrees of freedom to represent the relationship between canopy cover and temperature. The probability of selection of denser canopy increased significantly with temperature during summer for all four regions, where red lines indicated the 90% temperature percentiles of experienced temperature and the blue lines indicate the 10% temperature percentiles experienced temperature by region. Shaded bands represent a 95% confidence interval. Plots were created in the ‘ggplot2’ R package [94]

Male moose in all three populations (no males were collared in the Tanana population, see Table 1) selected for increased canopy cover as temperature increased (Fig. 3; S3). However, like with the females, the response to selection of thermal cover was most pronounced in the most northerly region (Koyukuk; β_%canopy1_ = 27.84, *p* < 0.001; β_%canopy2_ = 24.30, *p* < 0.001; Table 5) as well as the most southerly region (Susitna; β_%canopy1_ = 22.51, *p* < 0.001; β_%canopy2_ = 14.71, *p* < 0.001). The effect of canopy cover was reduced in the Innoko males (β_%canopy1_ = 13.02, *p* < 0.001; β_%canopy2_ = 8.50, *p* < 0.001), yet the results still revealed highly statistically significant results indicating moose selected for increased canopy cover as temperature increased.

**Table 5:** Best habitat selection models for male Alaska moose from the step-selection function analysis. Natural spline (sp) predictors, where percent canopy interacted with temperature, have coefficients estimated for each line segment. Numbers one and two in the spline predictors represent an individual line segment. All three populations had temperature-canopy interactions with two-line segments. In the Innoko population, elevation was collinear with distance-to-water and was thus excluded. All predictors were standardized by dividing by two times their standard deviation. Robust standard errors are reported

Predictor	Population		
	Koyukuk	Susitna	Innoko
	Coefficient (SE)	Coefficient (SE)	Coefficient (SE)
Elevation	−0.45 (0.37)	−1.11 (0.28)^b^	NA
sp(Percent Canopy ^a^ Temperature)1	27.84 (4.6)^b^	22.51 (5.5)^b^	13.02 (3.3)^b^
sp(Percent Canopy ^a^ Temperature)2	24.30 (4.1)^b^	14.71 (3.8)^b^	8.50 (2.4)^b^
Percent Canopy	−16.63 (2.9)^b^	−11.81 (3.1)^b^	− 17.60 (2.04)^b^
Solar Radiation Index	0.02 (0.03)	−0.005 (0.003)	− 0.12 (0.02)^b^
Distance-to-Water	0.34 (0.31)	−0.59 (0.11)^b^	−0.02 (0.33)

^a^0.05; **0.01; ^b^0.001

Additionally, male moose in the Susitna population showed increased selection of locations closer to water and, like their female counterparts, avoided areas of higher elevation (β_elevation_ = − 1.11, *p* < 0.001). Similarly, Innoko males showed avoidance for areas with increased topographical solar radiation exposure (β_SRI_ = − 0.12, *p* < 0.001), but these selection behaviors were independent of temperature.

## Discussion

Our results demonstrate that moose at the northern extent of their range altered habitat selection patterns in response to temperature. Across all populations and sexes, moose selected for denser canopy cover as temperature increased, which is consistent with previous studies [20, 56, 88], and our prediction that moose would select cooler locations as ambient temperature increased.

### Magnitude of selection response to temperature varied by sex and population

Our habitat selection results also demonstrated that the magnitude of moose selection for dense canopy cover at higher temperatures varied between populations and sexes (Figs. 2 and 3; S2 and S3; Tables 4 and 5). In two (Innoko and Susitna) of the three populations containing both male and female moose, females demonstrated a stronger selection response for denser canopy at higher temperatures than males. This may be linked to calving and nursing demands on female moose [79] who may more strongly select for denser canopy cover to avoid spending calories to thermoregulate using physiological mechanisms. However, we were unable to distinguish between females with and without calves in this study. This likely influenced our results as females accompanied by their calves tend to increase selection for areas that provide cover for predator avoidance [24, 43] and drastically change their movements both before and after parturition [81].

We also considered whether population differences in selection strength may be related to the availability of thermal cover between regions (i.e., a functional response) where animals alter their habitat selection based on habitat availability [3, 63]. However, our results cannot entirely be explained by a functional response in habitat selection for thermal cover. For example, the Koyukuk moose showed strong selection for thermal cover as temperature increased but also had the second lowest available canopy cover regionally (37.6%; S4). Thus, we do not think a functional response per se explains regional differences in the selection strength, rather we anticipate that it is likely a combination of environmental factors interacting in complex ways to create a suite of unique habitat differences across regions (S5). However, to fully understand functional responses in habitat selection one must also consider the different spatial scales of selection [38, 63], as such responses are often evaluated at the landscape or home range scale [30, 32, 33, 60]. Thus, the lack of functional response of moose to canopy cover in our study may be related to the fine-scale nature of our analytical framework and not an absence of a functional response of moose to thermal cover.

### Implications of habitat selection results within a changing climate

The consistent patterns of resource selection for thermal refugia under increasing temperatures found in this study may have important implications for moose resilience in arctic-boreal landscapes responding to increased temperatures from global climate change. For instance, landscape changes associated with wildfire are generally reducing canopy cover from coniferous species, and annual area burned in North American boreal systems doubled in the last half century [44], which is strongly linked to climate and annual weather patterns [37, 45]. Vegetation in interior Alaska now has less older spruce forests, the most common thermal refugia by moose, and a greater proportion of early successional vegetation than before 1990 [51]. Burn severity also plays a major role in how boreal forests recover after wildfire [26], where areas of low burn severity in black spruce stands tend to undergo self-replacement succession [39] and areas of high burn severity favor relay succession of deciduous species over black spruce because of increased exposure of mineral soil and reduced seedbank availability [40, 78]. For moose, such changes in habitat structure may provide new forage resources [4, 47], but also may limit the available thermal refugia needed for behavioral thermoregulation immediately after disturbance events prior to vegetation regeneration, or in late spring (March–April) prior to budburst when moose have not yet shed their winter coats.

### Limitations and future work

Our results showed moose did not select for areas closer to water as temperature increased, which differ from previous observations where moose sought wetland or riparian areas to thermoregulate [76, 80]. We believe our results differed due to the spatial resolution (30 m grid cell size) used to represent this behavioral strategy. This restricted detection of smaller aquatic microhabitats important to moose. Unfortunately, no finer-scale map currently exists andlimited our ability to study selection for aquatic microhabitats, which may be especially relevant in flatter, more swamp-like areas such as the Tanana and Innoko regions.

Based on our results and limitations encountered, we make three broad recommendations for future work regarding animal behavioral thermoregulation. First, future work should investigate the vulnerability and resilience of arctic-boreal animals to structural habitat changes as forage resources increase and thermal cover decreases (e.g., [52, 88]). For example, recent work on Alpine ibex (*Capra ibex*) – another heat-sensitive ungulate – indicates that male ibex response to minimize heat stress comes at the expense of optimal foraging [9]. Unfortunately, we did not have a detailed forage quality or biomass model calibrated for our study areas and hesitated to use categorical land cover maps because of criticisms regarding their use [17]. In Alaska, there is not a wide distinction between shrub classes in landcover maps that would enable us to determine if selected shrub habitats correspond to palatable species and foraging behavior. For instance, “shrub” in most vegetative classifications does not distinguish between shade forages (*Salicaceae, Betula neoalaskana)* and shade only (*Alnus, B. nana*) species, which is critical for parsing selection behavior. Moose maximize energy intake in the hottest parts of summer, so selection for forage biomass and quality plausibly overrides thermal stress and predation risk for a time. However, we were unable to directly assess this tradeoff due to data limitations.

Second, we suggest testing for differences in female selection and movement relative to presence or absence of offspring. Such a distinction would connect nicely to calls to link behavior and movement to population outcomes [10, 59], especially when considering the thermal environment as survival and fitness often depend on the availability of suitable habitat to buffer against thermal extremes in a landscape [25].

Finally, a critical next step is to evaluate how habitat selection under thermal stress impacts individual fitness and population dynamics, as temperature plays an important role in limiting fecundity in other mammals [18, 93] including moose [50, 62]. This is especially important as population responses to climate change can vary dramatically. For instance, Joly et al. [42] found the influence of climate on caribou herds in Alaska was not uniform, instead, western populations increased in size while northwestern populations declined as a result of intensity changes in the Pacific Decadal Oscillation. Similarly, using detailed demographic information for caribou (*Rangifer tarandus*), red deer (*Cervus elaphus)*, and elk (*C. canadensis*) across the Northern Hemisphere, Post et al. [70] showed that that different population responses to climate varied in both direction and magnitude.

## Conclusion

The impacts of climate change in arctic-boreal regions increase landscape heterogeneity through processes such as increased wildfire intensity and area burned, which can significantly alter the thermal environment available to an animal. Despite recognizing the importance of thermal conditions to animals, there is a distinct lack of research on how animals might respond to climate driven changes in thermal refugia. Our regional assessment provides insight on how Alaska moose may respond to changes in ambient temperature, where statewide annual temperatures are averaging an increase of 0.4 °C per decade and summer temperatures are projected to increase 2–5 °C by midcentury [51]. Understanding habitat selection and movement patterns related to behavioral thermoregulation is a first step toward identifying areas capable of providing thermal relief for moose and other species impacted by climate change.

## Supplementary information

**Additional file 1: Supplementary 1:** Temperature Validation. **Supplementary 2:** Koyukuk males spline model results for elevation and temperature interaction. **Supplementary 3:** Interactive 3D plots of interaction between ambient temperature and canopy cover. **Supplementary 4:** Used-Available Tables of Covariates. **Supplementary 5:** Regional Habitat Features. Figure 1e: Regional variation in elevation. ANOVA results comparing regional variation in elevation show that all regions vary from each other statistically (F = 2705, *p* < 0.001). Figure 2e: Regional variation in ambient temperature. ANOVA results comparing regional variation in ambient temperature show that all regions vary from each other statistically (F = 2705, *p* < 0.001). With Tanana showing the highest temperatures, Innoko second, Koyukuk third, and Susitna fourth. Figure 3**:** Regional variation in cloud cover. ANOVA results show all regions vary from each other statistically (F = 1472, *p* < 0.001), except Koyukuk and Susitna. Table 1E**:** Regional variation in fixes occurring in the rain. Percent estimated proportionally comparing number of fixes in the rain to total number of fixes regionally.

## Data Availability

The GPS-telemetry data support the findings of this study are owned by Alaska Department of Fish and Game, National Park Service, and the Bureau of Land Management but restrictions apply to the availability of these data, which were used under license for the current study, and so are not publicly available.

## Abbreviations

ABoVE: Arctic-boreal vulnerability experiment
ADFG: Alaska Department of Fish and Game
AMAP: Arctic Monitoring and Assessment Programme
CLR: Conditional-logistic regression
Env-DATA: Environmental-data automated track annotation
GEE: Generalized estimating equations
GPS: Global positioning system
IPCC: Intergovernmental Panel on Climate Change
LOOCV: Leave-one-out cross validation
NARR: North American regional reanalysis
NASA: National Aeronautics and Space Administration
NOAA: National Oceanic and Atmospheric Administration
QIC: Quasi-likelihood under independence criterion
USGS: United States Geological Survey
RMSE: Root mean square error
RSAGA: R System for Automated Geospatial Analysis
S: Supplementary materials
Sp: Natural spline
SRI: Solar radiation index
SSF: Step selection function
