# Supplementary material for: Behavioral modifications by a large-northern herbivore to mitigate warming conditions
Source: Mov Ecol. 2020 Oct 15;8:39. doi: 10.1186/s40462-020-00223-9 (PMC7559473; doi:10.1186/s40462-020-00223-9)
Supplement: Supplementary file 1 — Additional file 1: Supplementary 1: Temperature Validation. Supplementary 2: Koyukuk males spline model results for elevation and temperature interaction. Supplementary 3: Interactive 3D plots of interaction between ambient temperature and canopy cover. Supplementary 4: Used-Available Tables of Covariates. Supplementary 5: Regional Habitat Features. Figure 1e: Regional variation in elevation. ANOVA results comparing regional variation in elevation show that all regions vary from each other statistically (F = 2705, p < 0.001). Figure 2e: Regional variation in ambient temperature. ANOVA results comparing regional variation in ambient temperature show that all regions vary from each other statistically (F = 2705, p < 0.001). With Tanana showing the highest temperatures, Innoko second, Koyukuk third, and Susitna fourth. Figure 3: Regional variation in cloud cover. ANOVA results show all regions vary from each other statistically (F = 1472, p < 0.001), except Koyukuk and Susitna. Table 1E: Regional variation in fixes occurring in the rain. Percent estimated proportionally comparing number of fixes in the rain to total number of fixes regionally. [file 40462_2020_223_MOESM1_ESM.zip › Supplementary Materials- Behavioral Modifications.docx]

**Supplementary 1: Temperature Validation**

Two moose populations (Koyukuk and Innoko) had temperature loggers on their GPS collars, enabling a comparison between these estimates and the temperature product (NARR) used in this study. GPS locations from the Koyukuk and Innoko populations were rarified to one randomly selected fix per individual per data to avoid issues with pseudoreplication. Using these rarified datasets, we regressed temperature estimates from NARR against recorded collar temperatures for both populations. To identify and remove outliers from temperature estimates, we used the Tukey method, which removed observations 1.5 times beyond the inner quartile range (Tukey, 1977). We used the Metrics package (Hamner & Frasco, 2018) to calculate the root mean square error (RMSE), which we used to asses bias of our regression estimates. Agreement between collar-based and NARR temperature estimates is moderate (Figure 1). These relationships are not as strong as we might have expected based on other studies comparing weather station data to collared estimated (*R^2^*=0.90 and 0.97, respectively Street et al., 2015; van Beest et al., 2012). However, the primary interest in incorporating temperature in these analyses is to determine how selection changes as a function of temperature through interaction terms with other covariates. To that end, our relatively large temperature pixels (32 km) represent an ambient, neighborhood temperature, allowing us to investigate how moose respond to ambient variation in temperature via fine-scale selection for environmental characteristics that are likely to create cooler micro-climates. Therefore we are confident the NARR temperature estimates used in this study are adequately representing ambient temperature for our purposes, while recognizing that no temperature data product will be completely accurate to on-the-ground conditions.


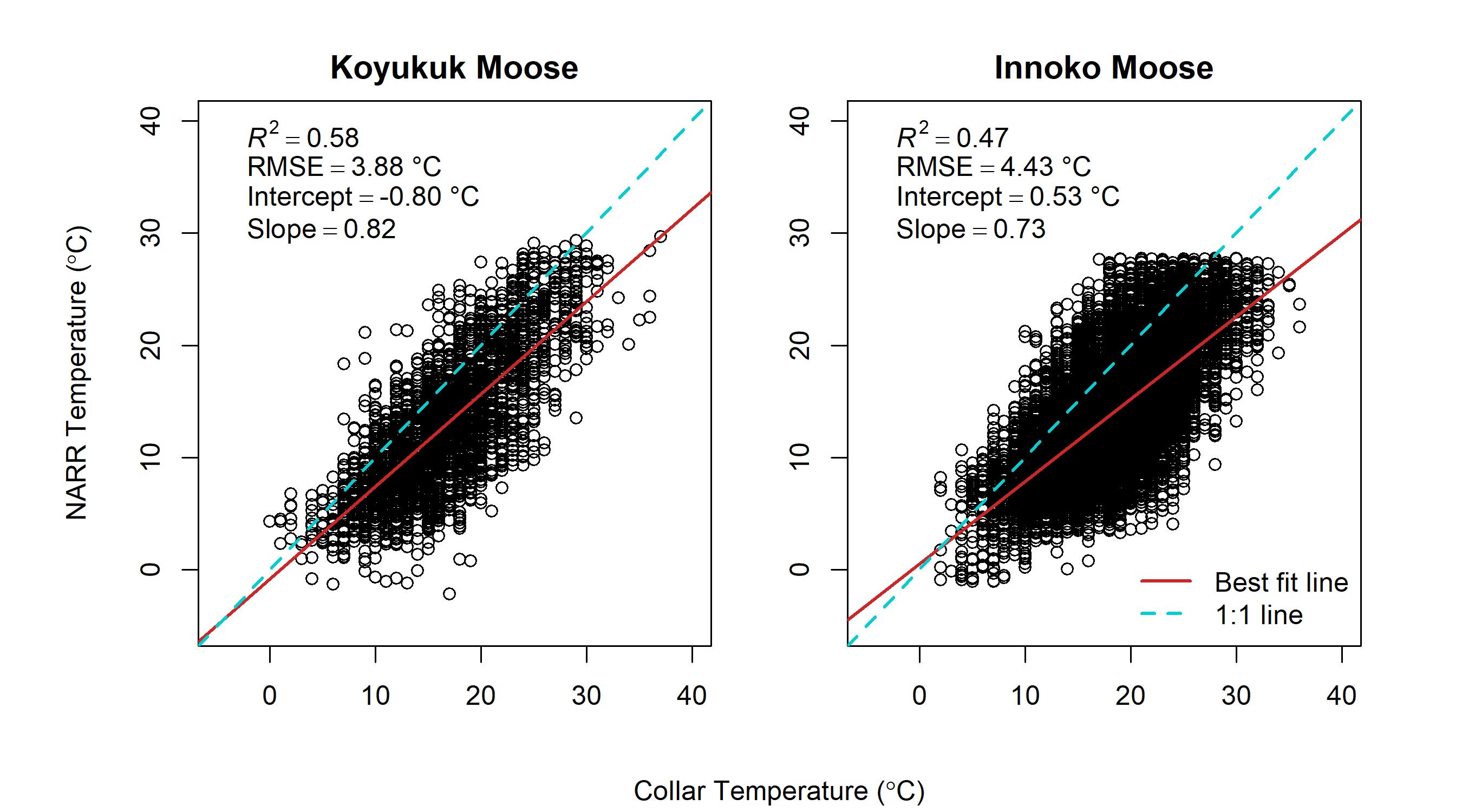


**Supplementary 2: Koyukuk males elevation*temperature spline model results (see RMarkdown html)**

**Supplementary 3: Interactive 3D plots of interaction between ambient temperature and canopy cover (see RMarkdown html)**

**Supplementary 4: Used-Available Tables of Covariates**

Used and available summaries by population for each covariate.

|  | **Females** | | | | | | | |
| --- | --- | --- | --- | --- | --- | --- | --- | --- |
| Predictors | **Koyukuk** | | **Susitna** | | **Innoko** | | **Tanana** | |
|  | Used  Mean (SD) | Available Mean (SD) | Used  Mean (SD) | Available Mean (SD) | Used  Mean (SD) | Available Mean (SD) | Used  Mean (SD) | Available Mean (SD) |
| **Elevation**  (meters) | 472.7 (204.4) | 476.8  (213.6) | 773.3 (204.7) | 784.9 (219.5) | 44.4  (39.8) | 44.5  (39.6) | 193.6  (118.2) | 193.5  (116.4) |
| **Percent Canopy**  (%) | 39.6  (32.2) | 31.9  (31.9) | 57.9  (29.6) | 51.8  (32.9) | 50.7  (33.9) | 46.1  (35.7) | 38.4  (28.3) | 37.4  (28.9) |
| **Solar Radiation Index**  (unitless) | 0.04  (0.6) | 0.03  (0.5) | -0.05  (0.69) | -0.05  (0.69) | -0.03  (0.66) | 0.006  (0.66) | -0.01  (0.5) | 0.02  (0.5) |
| **Distance to Water**  (meters) | 3969.8  (5885.7) | 3991.4  (5887.4) | 1373.3  (1017.3) | 1372.5  (1028.1) | 600.3  (1282.6) | 591.6  (1278.9) | 1427.7  (1113.4) | 1433.1  (1115.8) |

|  | **Males** | | | | | |
| --- | --- | --- | --- | --- | --- | --- |
| Predictors | **Koyukuk** | | **Susitna** | | **Innoko** | |
|  | Used  Mean (SD) | Available Mean (SD) | Used  Mean (SD) | Available Mean (SD) | Used  Mean (SD) | Available Mean (SD) |
| **Elevation**  (meters) | 520.0 (225.5) | 524.3  (238.1) | 805.4  (213.8) | 816.7  (224.6) | 54.9  (45.5) | 55.8  (47.9) |
| **Percent Canopy**  (%) | 35.6  (31.9) | 31.3  (31.5) | 54.27  (31.3) | 47.3  (34.4) | 44.8  (35.2) | 42.0  (36.2) |
| **Solar Radiation Index**  (unitless) | 0.02  (0.5) | 0.01  (0.5) | -0.06  (0.6) | -0.06  (0.6) | -0.01  (0.7) | 0.02  (0.7) |
| **Distance to Water**  (meters) | 2853.5  (2802.7) | 2854.3  (2799.7) | 1569.9  (1029.8) | 1553.6  (1051.3) | 1220.9 (2408.6) | 1225.7  (2397.4) |

**Supplementary 5: Regional Habitat Features**

We explored regional differences in habitat features that may explain our habitat such as elevation (Figure 1E), temperature (Figure 2E), cloud cover (Figure 3D), and precipitation (Table 1E). Elevation (m) data was sourced from the ArcticDEM (Porter, et al., 2018), while temperature (originally in Kelvin, but transformed into °C), cloud cover (%), and precipitation (binary: yes-no raining at time of fix) were sourced from NARR data (Mesinger et al., 2006) and annotated in Env-DATA (Dodge et al., 2013).


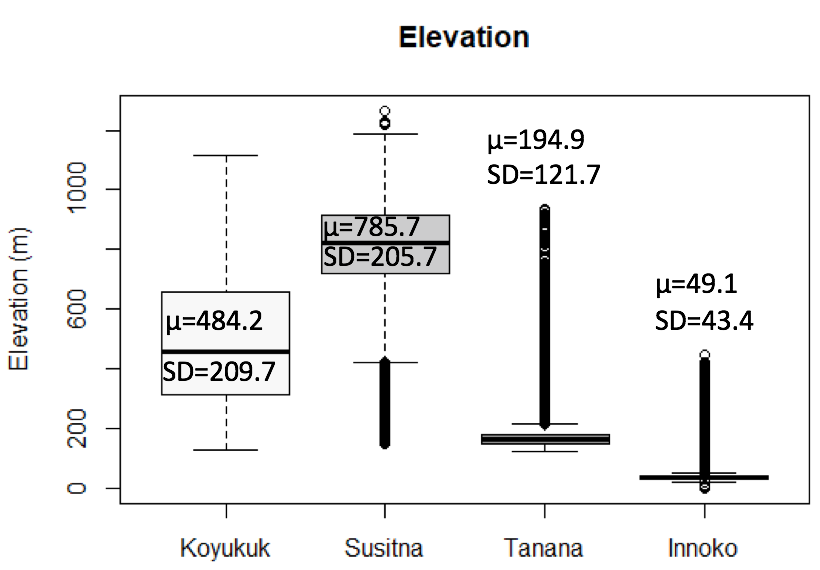


**Figure 1E: Regional variation in elevation.** ANOVA results comparing regional variation in elevation show that all regions vary from each other statistically (F=2705, p<0.001).


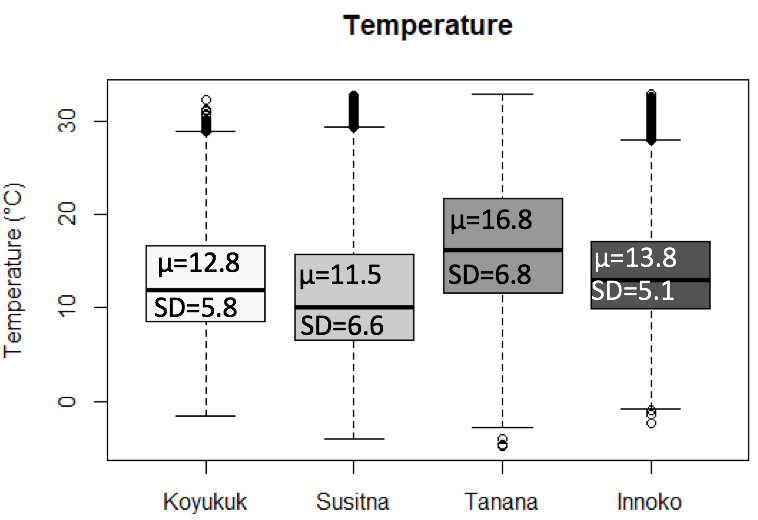


**Figure 2E: Regional variation in ambient temperature*.*** ANOVA results comparing regional variation in ambient temperature show that all regions vary from each other statistically (F=2705, p<0.001)*.* With Tanana showing the highest temperatures, Innoko second, Koyukuk third, and Susitna fourth.


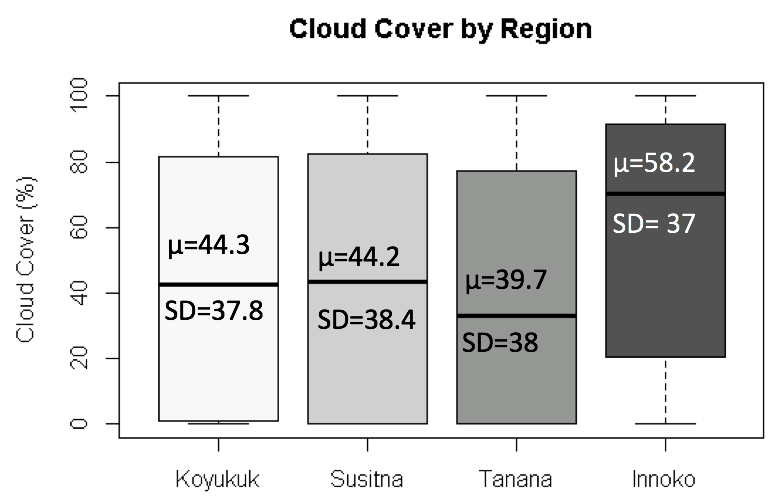


**Figure 3E: Regional variation in cloud cover*.*** ANOVA results show all regions vary from each other statistically (F=1472, p<0.001), except Koyukuk and Susitna.

**Table 1E:** **Regional variation in fixes occurring in the rain*.*** Percent estimated proportionally comparing number of fixes in the rain to total number of fixes regionally.

|  | Koyukuk | Susitna | Tanana | Innoko |
| --- | --- | --- | --- | --- |
| % fixes in the rain | 9.6% | 12.1% | 7.7% | 15.2% |
