# Supplementary material for: Behavioral modifications by a large-northern herbivore to mitigate warming conditions
Source: Mov Ecol. 2020 Oct 15;8:39. doi: 10.1186/s40462-020-00223-9 (PMC7559473; doi:10.1186/s40462-020-00223-9)
Supplement: Supplementary file 1 — Additional file 1: Supplementary 1: Temperature Validation. Supplementary 2: Koyukuk males spline model results for elevation and temperature interaction. Supplementary 3: Interactive 3D plots of interaction between ambient temperature and canopy cover. Supplementary 4: Used-Available Tables of Covariates. Supplementary 5: Regional Habitat Features. Figure 1e: Regional variation in elevation. ANOVA results comparing regional variation in elevation show that all regions vary from each other statistically (F = 2705, p < 0.001). Figure 2e: Regional variation in ambient temperature. ANOVA results comparing regional variation in ambient temperature show that all regions vary from each other statistically (F = 2705, p < 0.001). With Tanana showing the highest temperatures, Innoko second, Koyukuk third, and Susitna fourth. Figure 3: Regional variation in cloud cover. ANOVA results show all regions vary from each other statistically (F = 1472, p < 0.001), except Koyukuk and Susitna. Table 1E: Regional variation in fixes occurring in the rain. Percent estimated proportionally comparing number of fixes in the rain to total number of fixes regionally. [file 40462_2020_223_MOESM1_ESM.zip › Supplementary2 - Behavioral Modifications.html]

Appendix B: Koyukuk Males Elevation Spline Model


# Appendix B: Koyukuk Males Elevation Spline Model

### The best spline interaction model for the Koyukuk males came from temperature interacted with elevation with three degrees of freedom. It showed a greater model fit compared to both the base model (delta QIC= -169) and the percent canopy interacted with temperature spline model (delta QIC= -115); the elevation spline model also showed the largest improvement in predictive ability of any model considered (delta LOOCV= +18%). Therefore, we present this model as well, even though it was not discussed in detail in the main text of this manuscript. However, although this model appears to improve fit and predictive ability, results from the conditional logistic regression model show that elevation interacted with temperature is not a statistically significant predictor of male moose selection behaviors. This is likely due to individual variation present in this population, which also contains the smallest number of moose of any population considered in this study (N=11).

```
## Call:
## coxph(formula = Surv(rep(1, 43384L), used) ~ ns(elevation.std * 
##     surface.temp, 3) + elevation.std + percent.canopy.std + sri.std + 
##     distance.to.h20.100.std + cluster(ID_year) + strata(stratum), 
##     data = koyu.bulls.summer, robust = T, method = "breslow")
## 
##   n= 43324, number of events= 3942 
##    (60 observations deleted due to missingness)
## 
##                                            coef  exp(coef)   se(coef)
## ns(elevation.std * surface.temp, 3)1 -1.529e+01  2.295e-07  9.111e+00
## ns(elevation.std * surface.temp, 3)2 -4.380e+01  9.513e-20  1.919e+01
## ns(elevation.std * surface.temp, 3)3 -4.842e+01  9.408e-22  1.900e+01
## elevation.std                         1.671e+01  1.803e+07  7.668e+00
## percent.canopy.std                    4.003e-01  1.492e+00  4.412e-02
## sri.std                               2.686e-02  1.027e+00  3.362e-02
## distance.to.h20.100.std              -3.506e-01  7.042e-01  2.576e-01
##                                       robust se      z Pr(>|z|)    
## ns(elevation.std * surface.temp, 3)1  1.435e+01 -1.065    0.287    
## ns(elevation.std * surface.temp, 3)2  3.034e+01 -1.444    0.149    
## ns(elevation.std * surface.temp, 3)3  3.013e+01 -1.607    0.108    
## elevation.std                         1.220e+01  1.369    0.171    
## percent.canopy.std                    8.560e-02  4.676 2.93e-06 ***
## sri.std                               2.894e-02  0.928    0.353    
## distance.to.h20.100.std               3.870e-01 -0.906    0.365    
## ---
## Signif. codes:  0 '***' 0.001 '**' 0.01 '*' 0.05 '.' 0.1 ' ' 1
## 
##                                      exp(coef) exp(-coef) lower .95
## ns(elevation.std * surface.temp, 3)1 2.295e-07  4.356e+06 1.403e-19
## ns(elevation.std * surface.temp, 3)2 9.513e-20  1.051e+19 1.424e-45
## ns(elevation.std * surface.temp, 3)3 9.408e-22  1.063e+21 2.124e-47
## elevation.std                        1.803e+07  5.545e-08 7.418e-04
## percent.canopy.std                   1.492e+00  6.701e-01 1.262e+00
## sri.std                              1.027e+00  9.735e-01 9.706e-01
## distance.to.h20.100.std              7.042e-01  1.420e+00 3.298e-01
##                                      upper .95
## ns(elevation.std * surface.temp, 3)1 3.754e+05
## ns(elevation.std * surface.temp, 3)2 6.357e+06
## ns(elevation.std * surface.temp, 3)3 4.167e+04
## elevation.std                        4.384e+17
## percent.canopy.std                   1.765e+00
## sri.std                              1.087e+00
## distance.to.h20.100.std              1.504e+00
## 
## Concordance= 0.575  (se = 0.015 )
## Likelihood ratio test= 304  on 7 df,   p=<2e-16
## Wald test            = 168.5  on 7 df,   p=<2e-16
## Score (logrank) test = 239.3  on 7 df,   p=<2e-16,   Robust = 15.79  p=0.03
## 
##   (Note: the likelihood ratio and score tests assume independence of
##      observations within a cluster, the Wald and robust score tests do not).
```

### Figure 1B: Conditional probability of selection of spline-based elevation as a function of temperature for the Koyukuk males in summer months (June-August). We used a natural spline with three degrees of freedom to represent the interaction between elevation and temperature. Red coloring indicate warmer temperatures, whereas blue indicates cooler temperatures. Click and hold on the figure to adjust viewing perspective.
