# Supplementary material for: Behavioral modifications by a large-northern herbivore to mitigate warming conditions
Source: Mov Ecol. 2020 Oct 15;8:39. doi: 10.1186/s40462-020-00223-9 (PMC7559473; doi:10.1186/s40462-020-00223-9)
Supplement: Supplementary file 1 — Additional file 1: Supplementary 1: Temperature Validation. Supplementary 2: Koyukuk males spline model results for elevation and temperature interaction. Supplementary 3: Interactive 3D plots of interaction between ambient temperature and canopy cover. Supplementary 4: Used-Available Tables of Covariates. Supplementary 5: Regional Habitat Features. Figure 1e: Regional variation in elevation. ANOVA results comparing regional variation in elevation show that all regions vary from each other statistically (F = 2705, p < 0.001). Figure 2e: Regional variation in ambient temperature. ANOVA results comparing regional variation in ambient temperature show that all regions vary from each other statistically (F = 2705, p < 0.001). With Tanana showing the highest temperatures, Innoko second, Koyukuk third, and Susitna fourth. Figure 3: Regional variation in cloud cover. ANOVA results show all regions vary from each other statistically (F = 1472, p < 0.001), except Koyukuk and Susitna. Table 1E: Regional variation in fixes occurring in the rain. Percent estimated proportionally comparing number of fixes in the rain to total number of fixes regionally. [file 40462_2020_223_MOESM1_ESM.zip › Supplementary3- Behavioral Modifications.html]

Supplementary 3


# Supplementary 3

### Conditional probability of selection of spline-based thermal cover as a function of temperature for Alaska moose (Alces alces gigas) by population in summer months (June-August). We used natural splines with two to three degrees of freedom to represent the interaction between canopy cover and temperature. The probability of selection of denser canopy increased significantly with temperature during summer for both sexes and all populations. Red coloring indicate warmer temperatures, whereas blue indicates cooler temperatures. Click and hold on any figure to adjust viewing perspective.

# Female Moose

# Male Moose
